# Hyperoside flavonoids protect against malathion-induced mitochondrial toxicity in the differentiated SH-SY5Y cells

**DOI:** 10.1007/s00210-025-04379-2

**Published:** 2025-06-25

**Authors:** Ekramy M. Elmorsy, Huda A. Al Doghaither, Ayat B. Al-Ghafari, Manal S. Fawzy, Eman A. Toraih, Noha M. Abd El-Fadeal

**Affiliations:** 1 Center for Health Research, Northern Border University, Arar, 73213 Saudi Arabia; 2 Department of Biochemistry, Faculty of Science, King Abdulaziz University, Jeddah, 21589 Saudi Arabia; 3 Experimental Biochemistry Unit, King Fahd Medical Research Center, King Abdulaziz University, Jeddah, 21589 Saudi Arabia; 4 Department of Surgery, School of Medicine, Tulane University, New Orleans, LA 70112 USA; 5 Department of Cardiovascular Perfusion, Interprofessional Research, College of Health Professions, Upstate Medical University, Syracuse, NY 13210 USA; 6 Genetics Unit, Department of Histology and Cell Biology, Suez Canal University, Ismailia, 41522 Egypt; 7 Medical Biochemistry and Molecular Biology Department, Faculty of Medicine, Suez Canal University, Ismailia, 41522 Egypt; 8 Biochemistry Department, Ibn Sina National College for Medical Studies, Jeddah, 22421 Saudi Arabia

**Keywords:** Differentiated SH-SY5Y cells, Hyperoside, Malathion, Mitochondrial toxicity, Neuroprotection, Oxidative stress

## Abstract

Malathion (MAL), an organophosphorus pesticide, is known to induce mitochondrial toxicity in neuronal cells, contributing to neurodegenerative processes. This study aims to investigate the protective effects of hyperoside (HYP), a flavonoid, against mitochondrial dysfunction induced by MAL in differentiated SH-SY5Y cells. Differentiated human neuroblastoma cell lines were treated with various concentrations of MAL (0.01 to 100 mM) and HYP (10 to 40 µM). Cell viability was assessed using MTT and BrdU assays, while mitochondrial function was evaluated through ATP production, mitochondrial membrane potential (MMP), oxygen consumption rates (OCR), mitophagy-related proteins (PARKIN and PINK1) evaluation, and expression of key mitochondrial genes (i.e., *ND1*/5, *Cy.b*, *CO1*, and *ATP 6/8*). Bioinformatics analyses were also employed to identify the pathways impacted by MAL exposure, which revealed disruptions in immune responses, apoptosis regulation, and mitochondrial function. MAL treatment resulted in significant concentration-dependent cytotoxicity and reduction in cell viability (*p* < 0.001). HYP treatment notably increased cell viability to 115.8 ± 3.5% and 130.1 ± 3.1% of the control cells’ viability at 20 and 40 µM concentrations, respectively. The cotreatment with HYP effectively restored mitochondrial function by increasing ATP levels and mitochondrial membrane potential (MMP), while also enhancing oxidative capacity (OCR). Importantly, HYP mitigated MAL-induced oxidative stress and apoptosis, restoring levels of PARKIN and PINK1 proteins, which are crucial for mitophagy. Additionally, HYP significantly enhanced the expression of mitochondrial genes involved in the electron transport chain in MAL-treated cells. These findings indicate that HYP provides significant protective effects against MAL-induced mitochondrial toxicity in differentiated SH-SY5Y cells, suggesting its potential as a therapeutic agent for mitigating pesticide-related neurotoxicity. Further research on HYP may enhance our understanding of its protective mechanisms and therapeutic applications in neurodegenerative diseases.

**Supplementary Information:**

The online version contains supplementary material available at 10.1007/s00210-025-04379-2.

## Introduction

Pesticides play a crucial role in modern agriculture, effectively managing weeds, diseases, and pests. Their use has significantly boosted agricultural productivity (Jogaiah et al. 2007; Sudisha et al. 2010; Dubey et al. 2015). Among the various pesticides employed in India, organophosphorus (OP) chemicals are prevalent due to their perceived low toxicity to plants, minimal environmental impact, and limited effects on human health and food safety (Gao et al. 2009; Satapute et al. 2019).

Malathion (MAL), one of the earliest OP insecticides introduced in the 1950 s, is widely used for agricultural and public health purposes, including mosquito control (Paul et al. 2015). Classified as a Class III (somewhat hazardous) chemical, MAL is considered potentially carcinogenic. It can metabolize to malaoxon (MO) in the liver—an acetylcholinesterase (AChE) inhibitor that is approximately 100 times more potent than MAL, exhibiting mammalian IC30 and IC50 values around 0.1 and 2.4 µM, respectively (Krstić et al. 2008; Tarhoni et al. 2008). Both acute and chronic exposure to MAL has been linked to alterations in the expression and activity of enzymes and proteins that contribute to neurobehavioral effects and developmental neurotoxicity. These include mechanisms such as oxidative stress, neuroinflammation, and apoptosis (Fortunato et al. 2006; dos Santos et al. 2016; El-Harouny et al. 2017; Akbel et al. 2018).

Exposure to environmental or domestic pesticides, including OPs like MAL, is proposed as a risk factor for neurodegenerative disorders such as Alzheimer’s and Parkinson’s diseases (Sánchez-Santed et al. 2016; Sarailoo et al. 2022). Although the etiology of these disorders is not fully understood, mitochondrial damage and oxidative stress are consistently implicated as key factors in disease pathogenesis and progression (Jurcau 2021). This underscores the need for protective strategies against pesticide-induced mitochondrial toxicity.

Hyperoside (HYP), a flavonoid glycoside found in various plant families such as “Labiatae, Ericaceae, Rosaceae, Hypericaceae, and Campanulaceae,” exhibits numerous biological activities, including anti-inflammatory and antioxidant effects (Zhang and Zhang 2019). HYP’s neuroprotective properties have been demonstrated in models of depression, epilepsy, Parkinson’s-related disorders, and cerebral ischemia (Kwon et al. 2019; Cao et al. 2020; Song et al. 2022; Hong et al. 2023). However, its potential protective effects against malathion-induced mitochondrial toxicity have not been thoroughly investigated.

Despite the known risks of malathion-induced mitochondrial toxicity and the promising protective properties of hyperoside in various neurological models, there is a lack of research on its efficacy in counteracting malathion’s toxic effects on mitochondria. Hence, this study aims to fill this gap by investigating the protective role of hyperoside against malathion-induced mitochondrial toxicity using differentiated SH-SY5Y cells as an experimental model. In addition to biochemical assays, bioinformatics analyses were employed to identify molecular pathways affected by malathion exposure. Mitochondria play a crucial role in cellular health and disease, and mitochondrial disruption is central to cytotoxic mechanisms such as oxidative damage, apoptosis, and inflammation (Ott et al. 2007), key mechanisms implicated in neurodegeneration. In this context, this study examines the potential of hyperoside to mitigate malathion-induced mitochondrial damage in differentiated neuroblastoma cells.

## Materials and methods

### Preparation of MAL and HYP stock solutions

All chemicals were purchased from Sigma (St. Louis, MO, USA) unless specified otherwise. MAL was dissolved in dimethyl sulfoxide (DMSO), and HYP was dissolved in the culture medium.

### Cell culture

The SH-SY5Y cell line was obtained from the European Collection of Authenticated Cell Cultures (ECACC) (ECACC 94030304). Cells applied for the experiments are from passages 13 and 14 of the cell line. Cells were grown in a culture medium of 43.5% Eagle’s Minimum Essential Medium (EMEM) (M4655, Sigma-Aldrich, Poole, UK). The medium was supplemented with 43.5% Ham’s F12 nut mix (217,665–029, Gibco, Waltham, USA); 10% heat-inactivated Fetal Bovine Serum (FBS) (F9665, Sigma-Aldrich, Poole, UK); 1% MEM Non-Essential Amino Acid Solution (NEAA) (RNBF3937, Sigma, Poole, UK); 2 mM glutamine and 1% penicillin–streptomycin solution containing 10,000 IU penicillium and 10 mg/mL streptomycin (p/s) (P4333, Sigma-Aldrich, Poole, UK) in 25 or 75 cm^2^ flasks (Thermofisher scientific, Rochester, UK) at 37 °C with an atmosphere of 5% CO_2_ and 95% humidity. Cells were checked daily until they attained 80% confluence, after which the culture media was changed every other day.

### Differentiation of SH-SY5Y cells

Initially, SH-SY5Y cells were seeded in 25 cm^2^ flasks coated with poly-D-lysine hydrobromide (5 mg/mL). After the cells reached 60% confluency, they were cultured in a 10% FBS medium. The cells were then grown in SH-SY5Y media containing 10 µM all-trans retinoic acid (RA) and maintained in the dark for 3 days. After that, the media was discarded, and fresh media containing 80 nM 12-O-tetradecanoylphorbol-13-acetate (TPA) was added. The cells were then incubated for 3 days to induce differentiation. Differentiation was validated by ELISA’s higher levels of tyrosine kinase in the differentiated SH-SY5Y neurons using an Abcam commercial kit (Shipley et al. 2016). For further 72-h assays, the differentiation media were supplemented with Brain-Derived Neurotrophic Factor (BDNF) (50 ng/mL) to maintain the cells’ survival and maturation (Bilginer Kartal and Arslan Yildiz 2024).

### Determination of cytotoxicity and cellular viability of SH-SY5Y cells

The cytotoxic effect of MAL on the differentiated neurons was assessed using an MTT assay, following the procedures outlined in a prior publication (Elmorsy et al. 2014). A 96-well plate was used for cell subculturing, with 1 × 10^4^ cells seeded per well and an overnight incubation period. They received 72 h of treatment with either HYP at doses of 10, 20, and 40 µM or MAL at concentrations of 0.01, 0.1, 1, 10, or 100 mM. After adding MTT reagent to each well, the cells were incubated for 2 h. Each well was then filled with 100 µL of solubilizing reagent. A Dyne MRX microplate reader (Dyne Technologies, Chantilly, VA, USA) was used to measure the absorbance at 590 nm after an extra hour of incubation and 30 s of medium-speed shaking. In the experiment, each cell line underwent a minimum of three tests. MTT assay was repeated using MTT estimated E (2 mM) C50s and 0.2 µM concentrations in the presence and absence of coculture with HYP (20 or 40 µM), antioxidant GSH-R (10 µM), caspase-3 inhibitor z-VAD-fmk (200 µM), or mitochondrial enhancer Co-Q10 (1 µM).

### Cell proliferation assay (BrdU assay)

Following the company’s procedure (Millipore, MA, USA), BrdU proliferation assays were used to assess neuronal cell proliferation under experimental treatment conditions. Cells were seeded in 96-well plates at a density of 2 × 10^4^ cells per well and incubated until they reached 90% confluence. They were then treated with DEX at doses of 5 and 50 µM for 12 and 24 h. The medium was discarded after adding the “Bromodeoxyuridine (BrdU)” reagent to the wells for the final 2 h of exposure. Approximately 200 µl of fixing solution was added to each sample and kept at room temperature. After 30 min, the solution was aspirated, and the well plates were cleaned. A diluted anti-BrdU monoclonal antibody was given, followed by goat anti-mouse IgG. After washing, the plates were dried, and peroxidase substrate was added. An acid-stop solution was then added before measuring the absorbance. The TopCount plate reader (PerkinElmer, Ueberlingen, Germany) measured the absorbance at 450 nm. The experiment was repeated three times, subtracting the control well absorbance values from those of the DEX-treated samples.

### Bioenergetics assays

For the bioenergetic assays, cells were seeded and kept overnight for 80–90% confluence, then treated with HYP (20 or 40 µM) for 24 h in the presence or absence of MAL (0.2 or 2 mM).

#### Effect of MAL and HYP on intracellular ATP level

Cells were seeded on a 6-well plate at a density of 2 × 10^6^ and treated for the MTT assay. After 24 h, intracellular ATP levels were assessed using the CellTiter-Glo® Luminescent Cell Viability Assay (Promega, Cat. #G7570), adhering to the manufacturer’s protocol with modifications for a 6-well plate format. Cells were seeded in 6-well plates at a density of 2 × 10^5^ cells per well and incubated overnight to promote adhesion. Following treatment, the media were aspirated, and cells were washed once with phosphate-buffered saline (PBS) to remove residual media and serum components. Each well absorbed 500 µL of CellTiter-Glo® reagent, composed of reconstituted lyophilized substrate and the supplied buffer. Plates were placed on an orbital shaker for 10 min at room temperature to ensure complete cell lysis and signal stability. Utilizing a plate reader (e.g., BioTek Synergy HTX) with an integration time of one second per well, 100-µL aliquots were transferred from lysates in microcentrifuge tubes to white opaque 96-well plates for luminescence detection. The total protein content of the residual lysate was quantified using the bicinchoninic acid (BCA) assay (Thermo Fisher Scientific). ATP levels were normalized to protein content and expressed as relative luminescence units per µg of protein. Each experiment was conducted in triplicate, and all results were subsequently adjusted by subtracting the background fluorescence recorded from blank wells containing only the reagent.

#### Effect of MAL and HYP on mitochondrial membrane potential

Utilizing a MitoTracker Green (MG) probe, the effect of MAL and HYP on the treated cells’ MMP was assessed. Cells were plated and treated for the ATP assay. At this point, the media were removed, and the plates were incubated at 37 °C for 30 min with a 50 nM MG staining solution. The cells were then washed and placed in fresh phosphate-buffered saline for fluorescence measurement using a TopCount PerkinElmer microplate reader with excitation and emission filters set at 490/515 nm.

#### Effect of MAL and HYP on mitochondrial complexes I (MCI) and III (MCIII) activity

MCI and MCIII activities were assessed following the methodology outlined by Spinazzi et al. (2012) and modified by Elmorsy et al. (2021) to be applicable to the plate’s reader. Mitochondria-enriched fraction and cell lysate were prepared, and rotenone and antimycin A were used as specific inhibitors for MCI and MCIII assays, respectively. Contents of the MCI and MCIII assay buffers’ components are shown in Table S1. Absorbance was read at 340 and 550 nm for MCI and MCIII, respectively, using a Dyne MRX micro-plate reader for every sample concentration. At least four experiments were carried out.

#### Effect of MAL and HYP on lactate production

Following the manufacturer’s instructions (Biovision Inc., Milpitas, CA, USA), cells were grown in a 6-well plate and treated as described in the previous sections. Twenty-four hours after exposure, the media were collected, and lactate levels were measured calorimetrically using a commercial lactate test kit (Catalog Number: K667-100). Absorbance was read at 570 nm. Each assay point was carried out three times. Readings were normalized to the protein content of the samples after subtracting the readings from the blank wells (cell-free wells).

### Measuring the effect of MAL and HYP on the oxygen consumption rates (OCR)

Cells were plated in 6-well plates and treated with MAL and HYP for 24 h. After treatment, the cells were trypsinized, centrifuged, and resuspended in a modified Hank’s solution (Table S1), and then counted. Subsequently, the OCR was measured using Oxygen consumption electrodes (Clark oxygen electrodes, Rank Brothers, Bottisham, UK) under basal conditions for 10 min. The change in oxygen levels (ΔO2) was calculated over a 300-s period. Most changes in oxygen levels showed a linear slope, while for Azide, the slope was measured 60 s after its addition. Each drug concentration was tested at least 7 times to ensure reliable data.

### The effect of MAL and HYP on mitophagy in the treated cells

Cells were grown in a 6-well plate and treated as in the previous sections. Twenty-four hours after exposure, the effects of HYP and MAL on the mitophagy of the treated cells were assessed by examining the expression of PINK1 and PARKIN proteins using a commercial ELISA kit (My Bioscience and Abcam, respectively) following the manufacturers’ instructions, with cell lysates. After adding the stop solution, absorbance was read at 450 nm by a Dyne MRX microplate reader. Levels were estimated by substituting the estimated optical density (OD) values at 450 nm into the prepared standard curve data.

### The effect of MAL and HYP on pyruvate dehydrogenase (PDH) and alpha-ketoglutarate (α-KG)

The effects of HYP and MAL on both PDH and α-KG were assessed using colorimetric assays with commercial kits (Abcam) according to the manufacturer’s protocol. The PDH activity was measured by reducing NAD^+^ to NADH + H^+^, followed by the reduction of a reporter dye to produce a colored reaction product (yellow), which was read at an OD of 450 nm. The α-KG assay was based on its conversion to pyruvate, which is utilized to convert a nearly colorless probe to a readable product at 570 nm via an ELISA Dyne MRX microplate reader.

### Measuring the effect of MAL and HYP on mitochondrial swelling and permeation to K^+ ^and H^+^ ions

The impact of MAL and HYP on mitochondrial swelling and permeability to H^+^ and K^+^ ions was examined following the protocol outlined by Yu et al. (2018). Cells were grown in a 6-well plate and treated as in the previous sections. Twenty-four hours after exposure, a mitochondrial-enriched fraction was prepared, and the protein content was determined using a Bradford assay. Mitochondria were treated with solution A to evaluate the influence of MAL and HYP on mitochondrial swelling. To investigate the effects on H^+^ permeation, mitochondria (0.5 mg/ml) were suspended in solution B. Mitochondria were suspended in solution C to examine the impact on K^+^ permeation. Components of solutions A, B, and C are shown in Table S1. All these solutions were supplemented with MAL (0.2 or 2 mM) and HYP (20 or 40 µM) and were then incubated for 30 min. Absorbance was measured for 20 min at 540 nm and 25 °C using a multi-mode microplate reader (FLUOstar Omega). Decreased absorbance means increased mitochondrial swelling and permeation to both H^+^ and K^+^ ions.

### Measuring the effect of MAL and HYP on the mitochondrial membrane fluidity (MMF)

Cells were grown in a 6-well plate and treated as in the previous sections. Twenty-four hours after exposure, the mitochondria were isolated according to the method described by Spinazzi et al. (2012) to assess the impact on MMF using a TMA-DPH fluorescent probe, following the procedure outlined by Pérez-Hernández et al. (2017). Protein levels were measured using the Bradford assay. TMA-DPH (0.25 mM) was then added to the mitochondrial suspension (0.5 mg protein/ml) and incubated in the dark for 30 min with stirring. Subsequently, fluorescence polarization was measured at temperatures ranging from 10 to 60 °C using a PerkinElmer fluorescence spectrometer, with excitation at 365 nm and emission at 425 nm, to evaluate the thermotropic characteristics of the mitochondrial membranes.

### Measuring the effect of MAL and HYP on the mitochondrial membrane fatty acid (MFA) composition

Cells were grown in a 6-well plate and treated as in the previous sections. Twenty-four hours after exposure, mitochondria were isolated following the MMF experiment. Mitochondrial lipids were extracted using the method outlined by Oemer et al. (2018) to assess the impact of MAL and HYP on MFA composition. Subsequently, trans-esterification was conducted per Morrison and Smith (1964) using boron trifluoride (14% w/v in methanol). Gas chromatography with a flame ionization detector (FID) on a 30 m Omega wax column was employed to analyze MFA. Saturated fatty acids (palmitic and stearic) and unsaturated fatty acids (palmitoleic, oleic, arachidonic, linoleic, and docosahexaenoic acid) were quantified. Ultra-highly pure nitrogen served as the carrier gas at a 14 ml/min flow rate. The injector and detector temperatures were set at 250 °C, while the column temperature was programmed to increase from 180 to 240 °C at a rate of 5 °C/min. The MFA composition is presented in mol%.

### Measuring the effect of MAL and HYP on mitochondrial bioenergetic gene expression

The influence of MAL and HYP on the expression of genes encoding mitochondrial complexes was examined using quantitative polymerase chain reaction (qPCR). The cells were treated with MAL and HYP as in previous assays. Subsequently, total RNA was isolated following the Qiagen manufacturer’s protocol. Next, cDNA was synthesized via reverse transcription from 200 ng of RNA using a commercial reverse transcription kit. Previously published qPCR primer sequences were utilized for this analysis (Piechota et al. 2006) (Table 1). The thermocycling conditions were adjusted according to the protocol of Huang et al. (2020), and the qPCRs were carried out using a CHYP96 real-time system (Bio-Rad Laboratories Inc.). Glyceraldehyde-3-phosphate (*GAPDH*) gene expression was utilized as a widely used internal housekeeping reference gene (Sikand et al. 2012). The expression of genes of interest was determined and normalized to the expression of *GAPDH*. Subsequently, the gene expressions of the treated cells were presented as fold changes relative to the gene expressions of the control (i.e., non-treated) cells. The qPCR experiments were conducted in triplicate.

**Table 1:** The primer sequences used in the quantitative real-time polymerase chain reaction

Gene	Sense (5′−3′)	Antisense (5′−3′)
*ND1*	ACACTAGCAGAGACCAACCGAA	GGGAGAGTGCGTCATATGTTGT
*ND5*	CTATCTCGCACCTGAAACAAGC	GGTGGAGTAGATTAGGCGTAGG
*Cy.b*	TATTCGCCTACACAATTCTCCG	GCTTACTGGTTGTCCTCCGATT
*CO1*	TACGTTGTAGCCCACTTCCACT	GGATAGGCCGAGAAAGTGTTGT
*ATP 6/8*	CCATCAGCCTACTCATTCAACC	GCGACAGCGATTTCTAGGATAG
*GAPDH*	GACAGTCAGCCGCATCTTCT	GCGCCCAATACGACCAAATC

*ND1* NADH dehydrogenase subunit 1, *ND5* NADH dehydrogenase subunit 5, *Cy.b* cytochrome B, *CO1* cytochrome C oxidase subunit 1, *ATP 6/8* ATP synthase subunit 6/8, *GAPDH* glyceraldehyde-3-phosphate dehydrogenase

### Bioinformatic verification

A search was conducted on the “Comparative Toxicogenomics Database (CTD)” using https://ctdbase.org/ to explore how mitochondrial pathways are affected by MAL and HYP. Subsequently, the “STRING database at https://string-db.org/” (last accessed October 20, 2024) was accessed to analyze protein–protein interactions related to mitochondrial functions, specifically ATP production. The search was enriched by incorporating gene ontology and pathway analysis, demonstrating co-expressed proteins and their interaction scores, as obtained from the STRING.

### Statistical analysis

All statistical analyses were conducted using PRISM 5 (GraphPad Software Inc., San Diego, CA, USA). Nonlinear curve fitting statistics determined the EC_50_ for MTT and LDH assays. The influence of MAL and HYP on mitochondrial complex enzyme kinetics was evaluated using nonlinear regression analyses. A Michaelis–Menten model was applied to establish the enzymatic Km substrate concentration at half-maximal enzyme velocity. Group data were compared using a one-way analysis of variance (ANOVA) test with Tukey multiple comparisons post-test. For two groups, comparisons were made using an unpaired Student *t*-test, or a Mann–Whitney test was applied whenever appropriate. Statistical significance was defined as *p* < 0.05.

## Results

### MAL and HYP effect on the neuronal differentiated SH-SY5Y cells’ viability

**Fig. 1:**
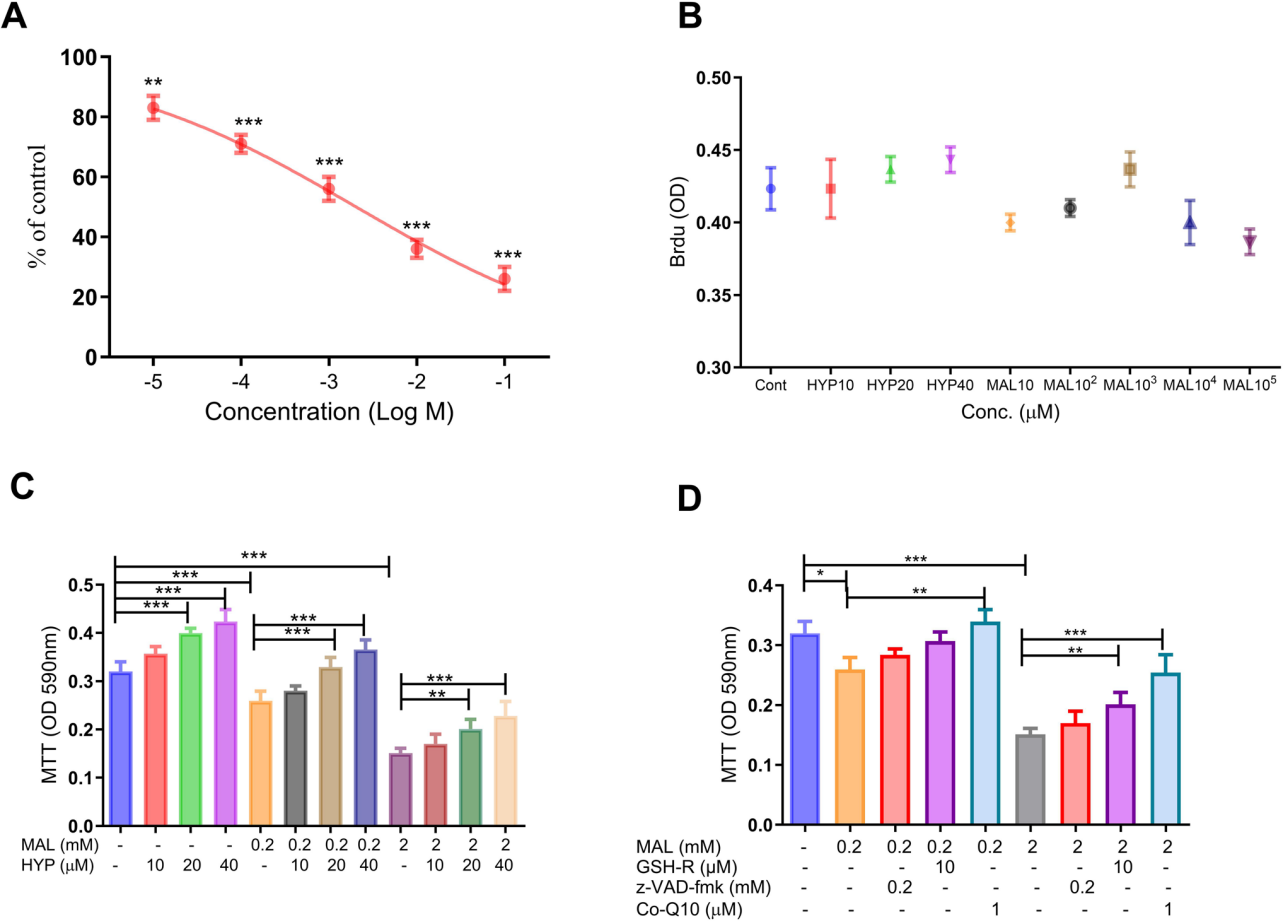
The effect of malathion (MAL) and hyperoside on the viability of the differentiated SH-SY5Y cells. **A** MTT assay revealed that MAL exhibited a concentration-dependent cytotoxic effect on the treated cells. **B** A BrdU assay revealed that MAL and HYP were cytotoxic to the tested cells without affecting cell proliferation. Histogram **C** showed that HYP significantly improved the cells’ viability at concentrations of 20 and 40 µM. In addition, cotreatment with HYP in both concentrations significantly mitigated the MAL-induced cytotoxic effect on the treated differentiated SH-SY5Y cells. The role of mitochondrial dysfunction, oxidative stress, and apoptosis in the observed cytotoxicity was evaluated using well-established inhibitors of these mechanisms (antioxidant GSH-R, caspase-3 inhibitor z-VAD-fmk, or mitochondrial enhancer Co-Q10). **D** Reduced glutathione and CoQ10 cotreatments with MAL (0.2 or 2 mM) were found to significantly improve cell viability, with a more pronounced effect of the mitochondrial enhancer CoQ10. Data were represented as means and standard deviations of at least 9 readings for each sample from the conducted triplicate of the assays, with at least 3 wells representing each treatment in each experiment. One-way ANOVA was used to assess the significance of differences among the groups, and Tukey’s multiple comparisons test was applied to determine the significance of differences among the individual groups. *means *p-*value < 0.05, **means *p-*value < 0.01, and ***means significance *p-*value < 0.001

The differentiated SH-SY5Y human neuroblastoma cells were treated with MAL at concentrations ranging from 0.01 to 100 mM, and their cytotoxicity was assessed after 24 h using MTT assays. The results indicated that MAL exhibited a concentration-dependent cytotoxic effect on the treated cells (Fig. 1A). Even at a concentration of 0.01 mM, MAL showed significant cytotoxicity (*p* < 0.001), reducing the cells’ ability to reduce the MTT dye to 81.3 ± 3.1% of the control cells’ capacity (Fig. 1A) . A BrdU assay revealed that MAL and HYP were cytotoxic to the tested cells without affecting cell proliferation (Fig. 1B). Additionally, the impact of HYP at concentrations of 10, 20, and 40 µM on viability was evaluated. Data showed that HYP significantly improved the cells’ viability at concentrations of 20 and 40 µM. In addition, cotreatment with HYP in both concentrations significantly mitigated the MAL-induced cytotoxic effect on the treated differentiated SH-SY5Y cells (Fig. 1C).

The role of mitochondrial dysfunction, oxidative stress, and apoptosis in the detected cytotoxicity was evaluated using well-known inhibitors of the mechanisms (antioxidant GSH-R (10 µM), caspase-3 inhibitor z-VAD-fmk (200 µM), or mitochondrial enhancer Co-Q10 (1 µM)). Firstly, the effect of MAL was on the cell viability at concentrations 0.2–2 mM, which reduced the cell viability to 73.2 ± 3.2 and 46.8 ± 3.4% of the control cells, respectively. Reduced glutathione and Co-Q10 cotreatments with MAL (2 mM) were found to significantly improve the cells’ viability by 64.5 ± 3.1 and 82.2 ± 2.9 times the control’s viability, with a more significant effect of the mitochondrial enhancer Co-Q10 in comparison to the antioxidant reduced glutathione (Fig. 1C).

HYP notably increased cell viability to 115.8 ± 3.5% and 130.1 ± 3.1% of the control cells’ viability at 20 and 40 µM concentrations, respectively. Interestingly, MAL (0.2 or 2 mM) cotreatment with HYP (20 or 40 µM) at concentrations 0.2 and 2 mM significantly counteracted the MAL-induced cytotoxic effect on the treated cells (Fig. 1D).

### MAL and HYP effects on the neuronal differentiated SH-SY5Y cells’ bioenergetics

**Fig. 2:**
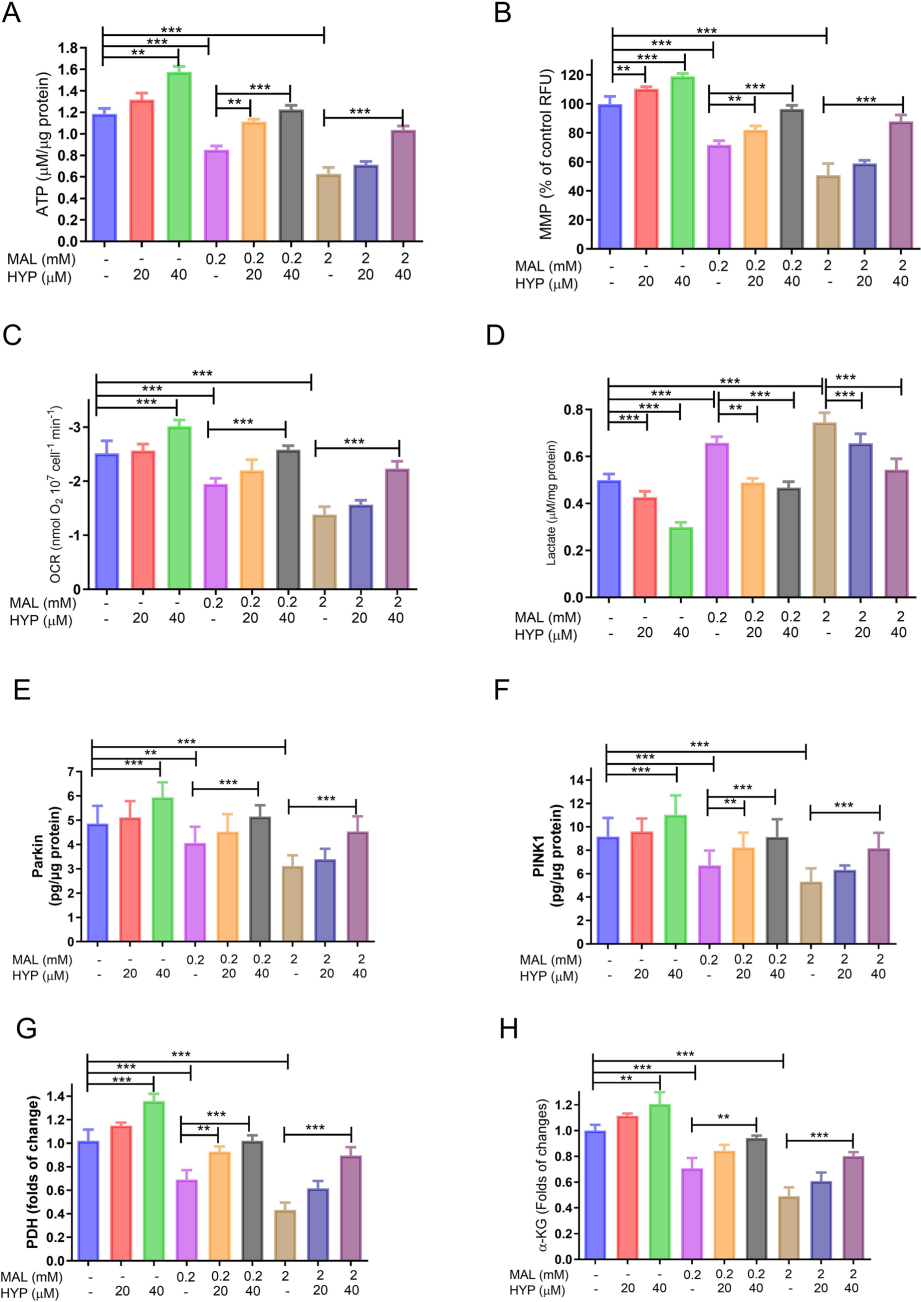
The effect of malathion (MAL) and hyperoside on the mitochondria of the differentiated SH-SY5Y cells. Histograms showed that MAL (0.2 or 2 mM) significantly decreased the levels of ATP (**A**), mitochondrial membrane potential (MMP) (**B**), and oxygen consumption rate (**C**). In parallel, MAL decreased the lactate production of the treated cells (**D**). Conversely, treatment with HYP (20 or 40 µM) significantly mitigated the MAL effect on the four bioenergetics parameters (**A**–**D**). Histograms also showed that MAL (0.2 mM) significantly decreased the mitophagy proteins PARKIN (**E**) and PINK1 (**F**) proteins and the activity of pyruvate dehydrogenase enzyme (PDH) (**G**) and alpha-ketoglutarate (α-KG) (**H**). This effect was antagonized by HYP treatment to variable extents. Data were represented as means and standard deviations of at least 9 readings for each sample from the conducted triplicate of the assays, with at least 3 wells representing each treatment in each experiment. One-way ANOVA was used to study the significance of differences among the groups, and Tukey’s multiple comparisons test was applied to specify the significance among the individual groups. *means *p-*value < 0.05, **means *p-*value < 0.01, and *** means significance *p-*value < 0.001

ATP assay revealed that intracellular ATP levels were significantly reduced to 71.2 ± 3.4% and 52.3 ± 2.9% of controls, respectively. Conversely, treatment with HYP at 20 and 40 µM significantly increased ATP to 110.8 ± 2.6% and 127.5 ± 3.2% of controls, respectively. Additionally, cotreatment of MAL and HYP at 40 µM significantly alleviated the effect of MAL on the ATP levels of the treated cells in a concentration-dependent manner (Fig. 2A).

The results of the MMP assay indicated that HYP (20 and 40 µM) significantly increased MMP to 111.5 ± 2.8% and 119.7 ± 2.9% of the control, respectively (*p* < 0.001). Additionally, MAL at 0.2 and 2 mM substantially reduced MMP to 71.4 ± 3.3 (*p* < 0.001), while co-administration of HYP at 20 µM with MAL (0.2 or 2 mM) restored MMP to 95.7 ± 3.1 and 84.3 ± 2.8 of the controls’ MMP, respectively (Fig. 2B).

Cellular bioenergetics were evaluated using OCRs’ polarographic assay. MAL significantly (*p* < 0.0001) reduced the OCRs of neuronal cells to approximately 76.3 ± 3.3 and 53.5 ± 3.8 at 0.2 mM and 2 mM concentrations, respectively. Conversely, HYP (40 µM) enhanced OCR to 122.1 ± 3.3 times that of the controls’ OCR. Samples cotreated with HYP (2 mM) exhibited notably higher OCR levels than those treated with MAL alone. Cotreatment with HYP (40 µM) substantially improved the OCRs of MAL-treated samples at 0.2 and 2 mM to 89.3 ± 4 and 79.3 ± 3.3% of the controls’ OCR, respectively (Fig. 2C).

Furthermore, data indicated that MAL (0.2 and 2 mM) significantly heightened lactate production (*p* < 0.001) due to activated anaerobic pathways of glucose metabolism to 129.7 ± 4.6 and 147.1 ± 3.9% of controls levels, respectively, while HYP cotreatment at 40 µM reduced lactate to 94.2 ± 3.3 and 105.2 ± 3.4 of the controls’ OCR, respectively (Fig. 2D).

PARKIN and PINK1 protein levels, markers for mitophagy, showed that MAL significantly reduced both protein levels. At a 2 mM concentration, MAL was shown to decrease PARKIN and PINK1 levels to 61.5 ± 2.8% and 54.9 ± 2.8% of the control levels. This effect was antagonized by HYP (40 µM) cotreatment, which increased protein levels to 91.5 ± 3.8% and 85.7 ± 2.3% of the control values (Fig. 2E and F).

The effect of MAL and HYP on the PDH enzyme, as the main point of entry of the Krebs cycle, was evaluated. Data indicated that MAL (0.2 and 2 mM) significantly reduced the enzyme activity level to 69.8 ± 3.2% and 45.2 ± 2.9% of the control levels, respectively, while HYP cotreatment at 40 µM significantly counteracted the effect of MAL on the tested enzyme. Also the effect of MAL and HYP on α-KG, as a main Krebs cycle intermediate, was estimated that MAL (0.2 and 2 mM) significantly lowered the enzyme activity level to 73.1 ± 2.8 and 49.6 ± 3.5% of controls levels, respectively, while HYP and MAL (0.2 and 2 mM) cotreatment at 40 µM significantly counteracted the effect of MAL on the tested enzyme and increased α-KG levels to 93.2 ± 2.8 and 79.5 ± 3.3% of controls levels, respectively (Fig. 2G and H).

**Fig. 3:**
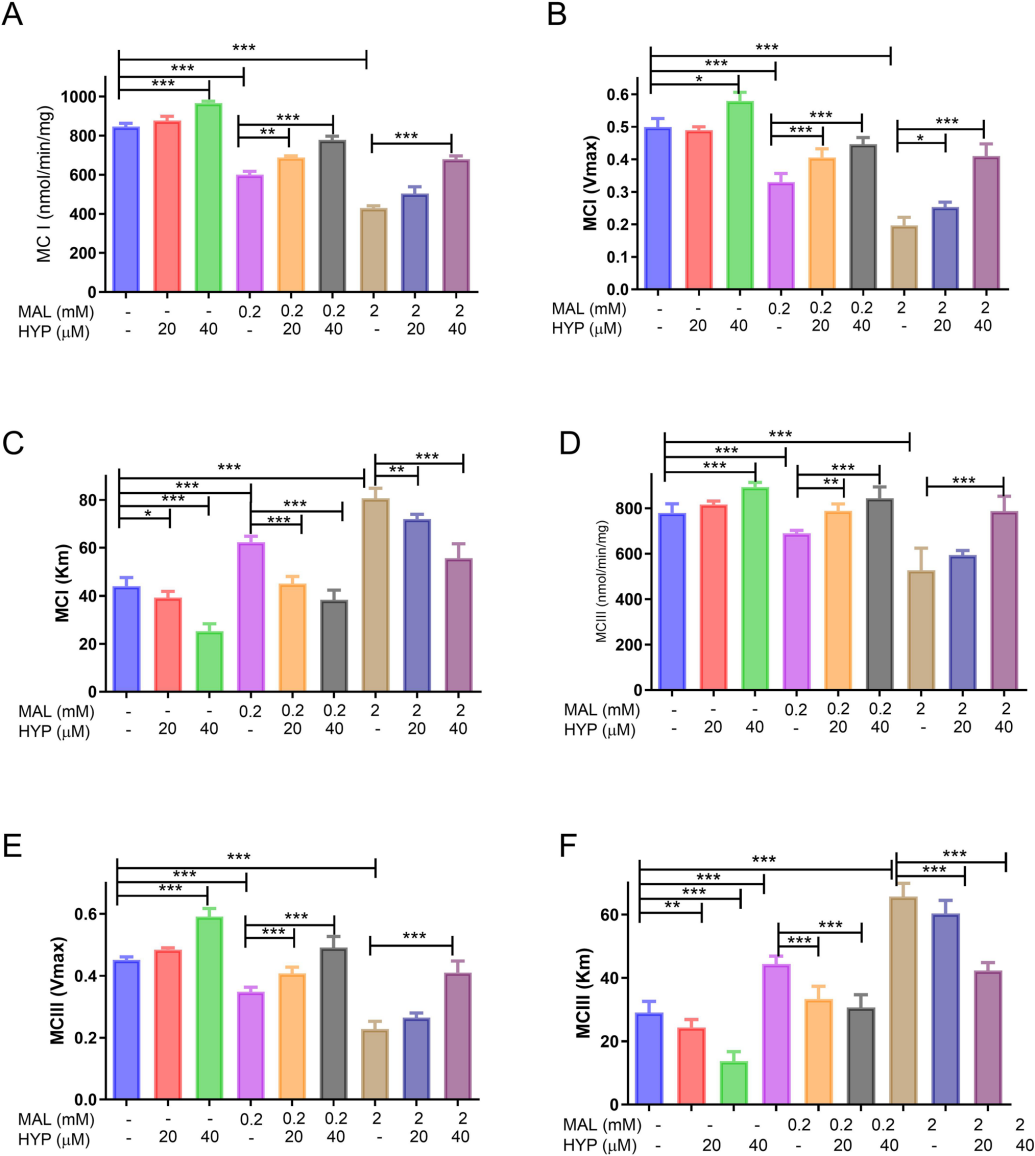
The effect of malathion (MAL) and hyperoside on the mitochondrial complexes (MCs) I and III of the differentiated SH-SY5Y cells. Following a 24-h exposure to HYP (20 and 40 µM), HYP significantly increased in MCI and its kinetic parameters, while MAL (0.2 and 2 mM) led to a decrease in MCI activity and its kinetic parameters. When cells were co-treated with MAL and HYP (20 and 40 µM), they showed significantly improved complex activity in comparison to MAL-treated samples (**A**–**C**). A similar effect of MAL and HYP was shown on the activities and kinetics of MCIII (**D**–**F**) to variable extents. Data were represented as means and standard deviations of at least 9 readings for each sample from the conducted triplicate of the assays, with at least 3 wells representing each treatment in each experiment. One-way ANOVA was used to study the significance of differences among the groups, with Tukey’s multiple comparisons test applied to specify the significance among the individual groups. *means *p-*value < 0.05, **means *p-*value < 0.01, and *** means significance *p-*value < 0.001

ATP production is regulated by oxidative phosphorylation linked to the mitochondrial electron transport system. To assess mitochondrial function, we measured the mitochondrial membrane potential and the activity of the mitochondrial enzymes complex I (MCI) and complex III (MCIII). Following a 24-h exposure to different concentrations of HYP (20 and 40 µM), we observed a significant increase in MCI activity to 106.3 ± 2.5 and 118.6 ± 1.5 times the control levels, respectively. In contrast, exposure to MAL (0.2 and 2 mM) led to a decrease in MCI activity to 73.2 ± 3.4 and 46.7 ± 3.1 of the control level, respectively. However, when cells were co-treated with MAL and HYP (20 and 40 µM), they showed significantly improved complex activity in comparison to MAL-treated samples (Fig. 3A).

In the case of MCI kinetics, our findings indicated that MAL treatment resulted in significantly higher Km and lower Vmax values. In contrast, HYP treatment led to concentration-dependent increases in Km and decreases in Vmax compared to the control samples. Additionally, HYP was observed to alleviate the effect of MAL on MCI enzyme kinetics (Fig. 3B, C).

Regarding the MCIII activity assay, our data revealed that HYP significantly improved the enzyme activity to 113.7 ± 3.6 at a 40 µM concentration. Conversely, MAL significantly reduced MCIII activity at both tested concentrations, 0.2 and 2 mM, to 88.4 ± 3.3 and 67.5 ± 5.6 of the control levels, respectively. Still, this effect was almost totally alleviated by HYP (40 µM) to levels around the control MCIII activity levels (Fig. 3D). The pattern of MCIII kinetics showed a similar trend to MCI kinetics, with a similar effect of MAL and HYP on Km and Vmax estimated values compared to control samples in a concentration-dependent manner (Fig. 3E, F).

**Fig. 4:**
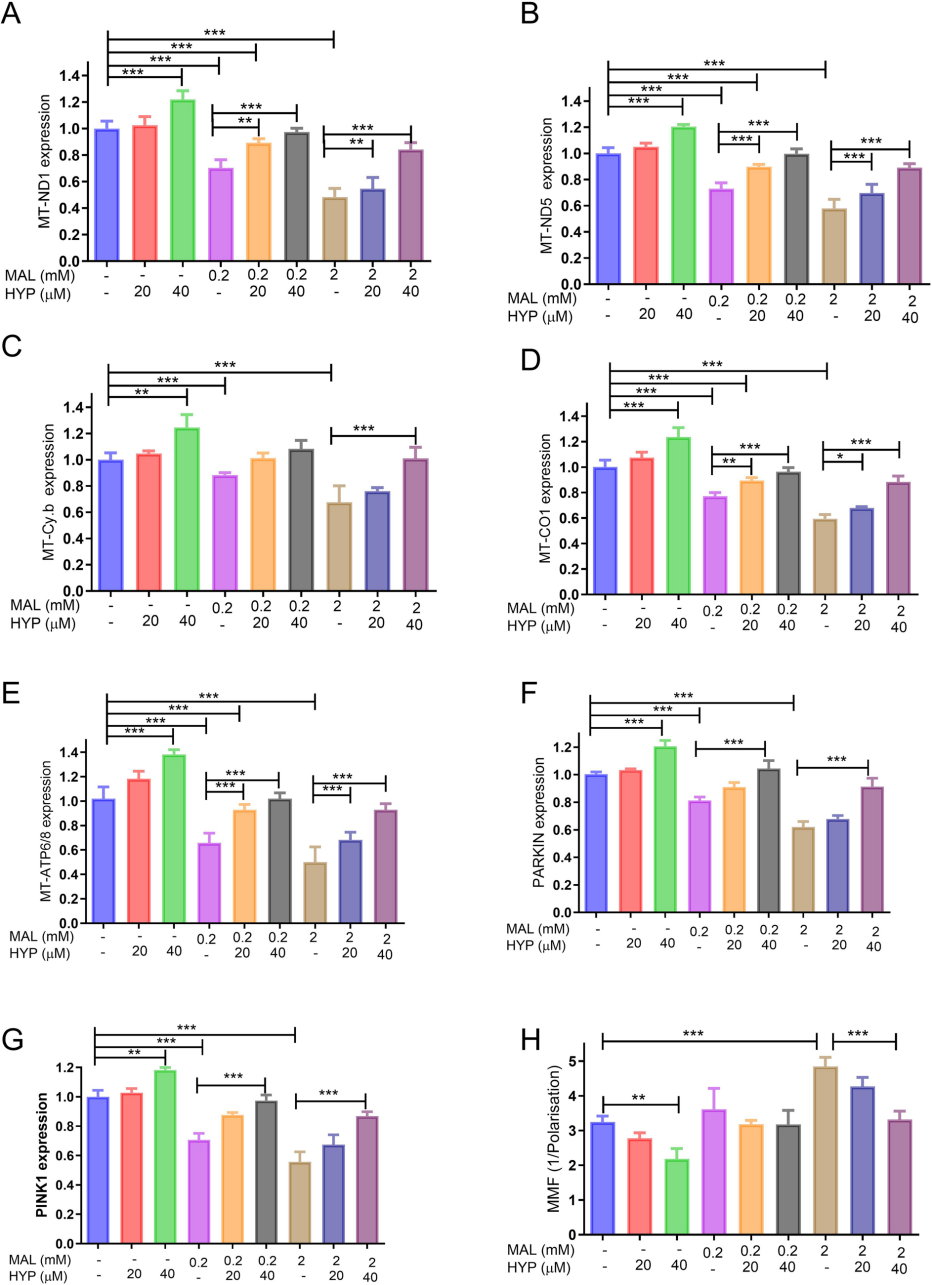
The effect of malathion (MAL) and hyperoside on the mitochondria-related genes and mitochondrial membrane fluidity (MMF). The qPCR data revealed that MAL at concentrations of 0.2 and 2 mM caused a significant decrease in the expression of mitochondrial genes related to the electron transport chain *ND1* (**A**), *ND5* (**B**), *Cy.b* (**C**), *CO1* (**D**), and *ATP6/8* (**F**), as well as mitophagy related genes *PARKIN* (**G**), and *PINK1* (**H**) with increased MMF (**I**) in a concentration-dependent manner. Conversely, HYP at 20 and 40 µM led to improved expression levels of these mitochondrial DNA-related genes and decreased MMF. MAL and HYP co-administered significantly counteracted the effects of MAL on mitochondrial gene expression and MMF (**A**–**I**). Data were represented as means and standard deviations of at least 9 readings for each sample from the conducted triplicate of the assays, with at least 3 wells representing each treatment in each experiment. One-way ANOVA was used to study the significance of differences among the groups, and Tukey’s multiple comparisons test was applied to specify the significance among the individual groups. *means *p-*value < 0.05, **means *p-*value < 0.01, and ***means significance *p-*value < 0.001

The qPCR data revealed that MAL at concentrations of 0.2 and 2 mM caused a significant decrease in the expression of mitochondrial genes related to the electron transport chain (*ND1*, *ND5*, *Cy.b*, *CO1*, *ATP6/8*, *PARKIN*, and *PINK1*) in a concentration-dependent manner. Conversely, HYP led to improved expression levels of these mitochondrial DNA-related genes at concentrations of 20 and 40 µM. Interestingly, when MAL was co-administered with HYP, it significantly mitigated the inhibitory effect of MAL on mitochondrial gene expression (Fig. 4A–G).

Furthermore, as the mitochondrial enzymes are primarily located on the mitochondrial membranes, the impact of MAL and HYP and their co-administration on MMF was evaluated. The data showed that treatment of the neuronal cells with MAL at concentrations of 0.2 and 2 mM significantly increased MMF levels to 112.8 ± 4.3 and 135.7 ± 2.4% of controls, respectively. Conversely, HYP at 20 and 40 µM significantly decreased MMF to 94.5 ± 3.3 and 104.5 ± 2.3% of controls, respectively. Additionally, the co-administration of MAL and HYP was found to effectively alleviate the impact of MAL on MMF levels of the treated neuronal cells in a concentration-dependent manner (Fig. 4F).

**Fig. 5:**
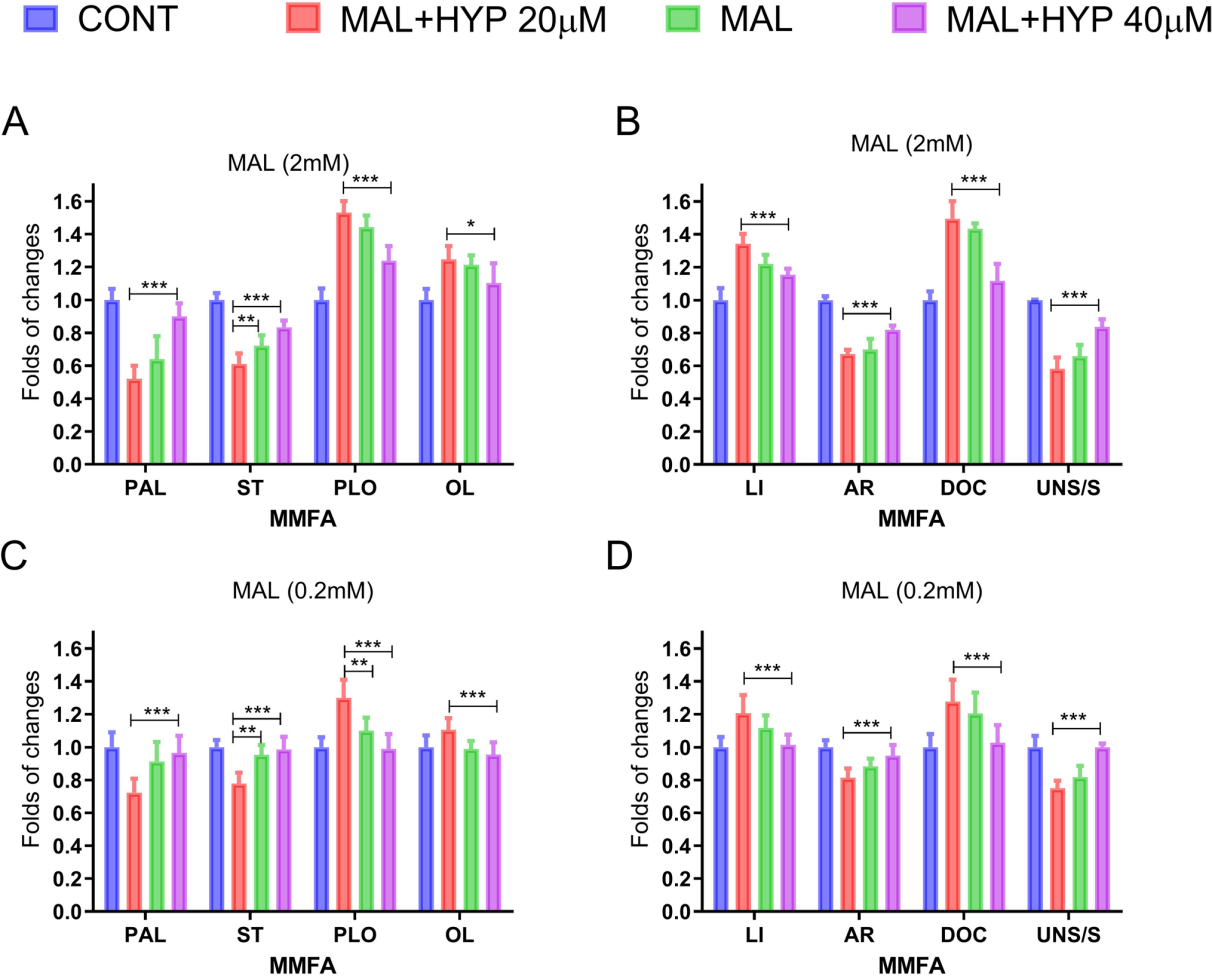
The effect of malathion (MAL) and hyperoside on the composition of mitochondrial membrane fatty acids (MMFAC). The histograms indicated that MAL and HYP caused noteworthy changes in the MMFAC of the treated human neuronal cells compared to the control samples. MAL led to an increase in unsaturated fatty acids; palmitoleic (PLO), oleic (OL), linoleic (Li), arachidonic (AR), and docosahexaenoic acid (DOC) composition and a decrease in saturated fatty acids; palmitic (PA) and stearic (ST) composition with increased unsaturated/saturated (US/S) fatty acids ratio, while cotreatment of MAL with HYP (40 µM) significantly mitigated the changes induced by MAL in MMFAC (**A**–**D**). Data were represented as means and standard deviations of at least 9 readings for each sample from the conducted triplicate of the assays, with at least 3 wells representing each treatment in each experiment. One-way ANOVA was used to study the significance of differences among the groups, with Tukey’s multiple comparison test applied to specify the significance among the individual groups. *means *p-*value < 0.05, **means *p-*value < 0.01, and ***means significance *p-*value < 0.001

The study also looked at the effects of MAL and HYP on MMFAC to better understand their impact. The results indicated that MAL and HYP caused noteworthy changes in the MMFAC of the treated human neuronal cells compared to the control samples. MAL led to an increase in unsaturated fatty acids composition and a decrease in saturated fatty acids composition, while cotreatment of MAL with HYP (40 µM) significantly mitigated the changes induced by MAL in MMFAC (Fig. 5A–D).

**Fig. 6:**
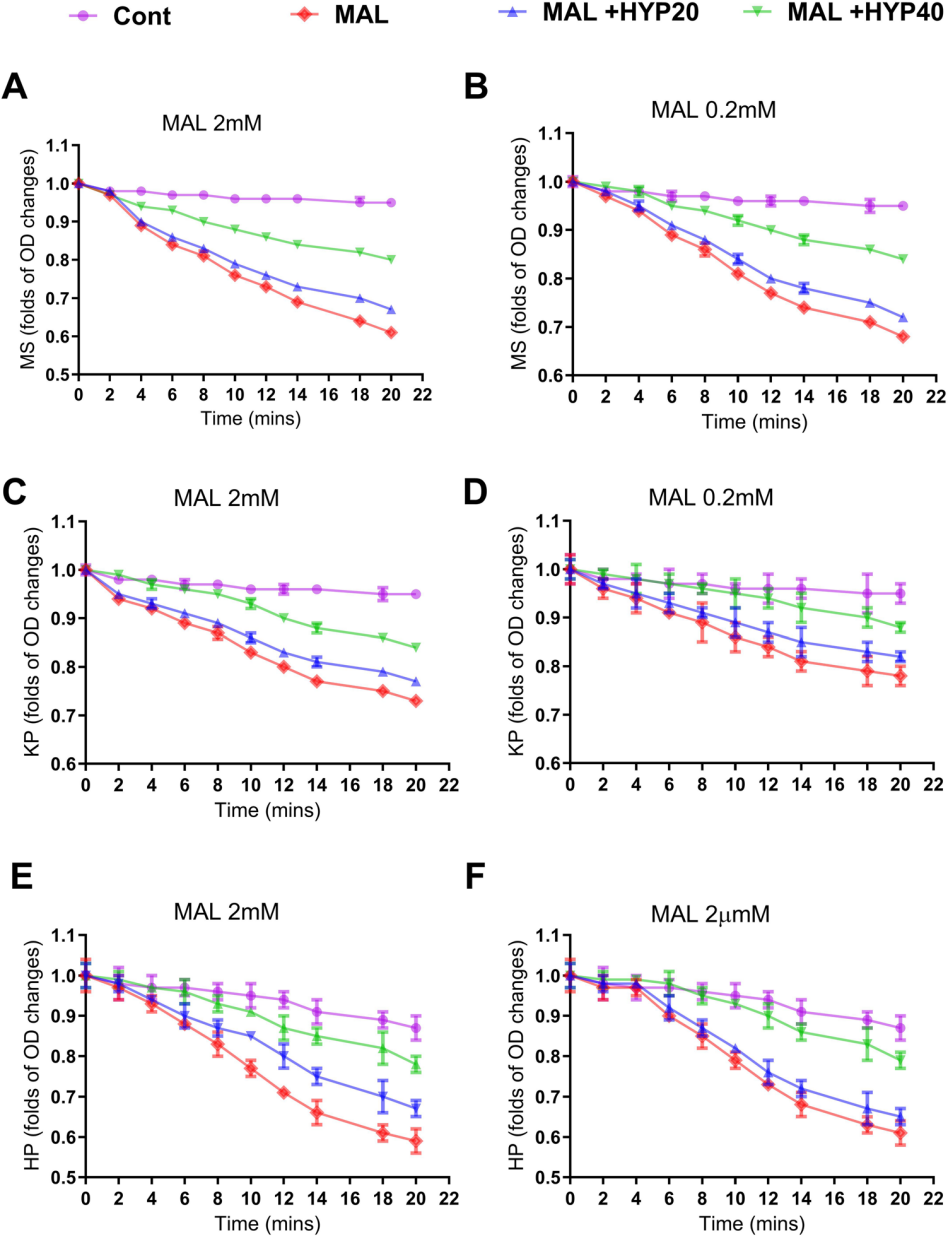
The effect of malathion (MAL) and hyperoside on the swelling and permeation of mitochondrial membranes to hydrogen (H^+^) and potassium (K^+^) ions. Assays showed that samples exposed to MAL (0, 2 or 2 mM) showed a gradual decrease in absorbance, indicating increased swelling (**A** and **B**) and altered permeation to K^+^ (**C** and **D**) and H^+^ (**E** and **F**). Cotreatment with HYP significantly reduced the impact of MAL at both tested concentrations (**A**, **F**). Data were represented as folds of changes from zero time point readings as means and standard deviations of at least 9 readings for each sample from the conducted triplicate of the assays, with at least 3 wells representing each treatment in each experiment. Two-way ANOVA indicated that the MAL and HYP significantly altered the tested parameters

Mitochondrial swelling is a well-known sign of mitochondrial dysfunction. As a result, we investigated the effects of the tested metals on mitochondrial swelling. The absorbance of the control group remained stable over time, indicating that the mitochondria maintained their integrity. However, the samples exposed to MAL showed a gradual decrease in absorbance to 70.2 ± 2.5% and 59.2 ± 2.3% of the control at concentrations of 0.2 and 2 mM, suggesting higher rates of mitochondrial swelling during the experiment. Cotreatment with HYP significantly reduced the impact of MAL at both tested concentrations (Fig. 6A, B).

Additionally, we evaluated mitochondrial membrane integrity concerning permeability to H^+^ and K^+^ ions. The slope decreased sharply in the MAL-exposed samples compared to the controls, indicating that MAL increases the permeability of the inner mitochondrial membrane to H^+^ and K^+^ ions. Cotreatment with HYP significantly mitigated the impact of MAL on the permeation of both ions at both tested concentrations (Fig. 6C–D, F). Two-way ANOVA was used to compare the data related to the effect of the MAL-tested concentrations on mitochondrial swelling and inner mitochondrial membrane permeation for H^+^ and K^+^ ions over the 20-min experimental fluorescence measuring time. Two-way ANOVA indicated that the MAL and HYP significantly altered the tested parameters. Details of the post-test are provided in Table S2. At the tested concentration, HYP at 20 and 40 µM concentrations did not considerably affect mitochondrial membrane swelling, K^+^, and H^+^ permeation (unpublished data).

The bioinformatics analysis indicates that exposure to MAL significantly impacts several key pathways, particularly those associated with immune responses, apoptosis, and metabolism, all of which are closely linked to mitochondrial function (Figure S1A). The most affected genes due to MAL toxicity are associated with apoptosis, oxidative stress, and mitochondrial dysfunction, as illustrated in Figure S1B. Notable genes implicated in MAL’s toxic effects include *ACHE*, *CAT*, *CASP3*, *TP53*, and *SOD2*. Additionally, data from toxicogenomics databases show strong correlations between malathion exposure and various diseases, including nervous system disorders, cancer, and metabolic conditions (Figure S1C). These findings reinforce the notion that mitochondrial damage and impaired mitophagy are critical mechanisms underlying MAL toxicity in the central nervous system.

Through our search of comparative toxicogenomics databases, we identified HYP as a promising compound for conditions such as brain ischemia, Alzheimer’s disease, and brain injuries (Figure S2A), all of which are linked to mitochondrial dysfunction. Due to its antioxidant and anti-inflammatory properties, HYP has demonstrated protective effects on mitochondrial function, effectively reducing damage caused by oxidative stress (Figure S2B). Additionally, its potential role in modulating apoptosis and inflammatory pathways further underscores its therapeutic value.

**Fig. 7:**
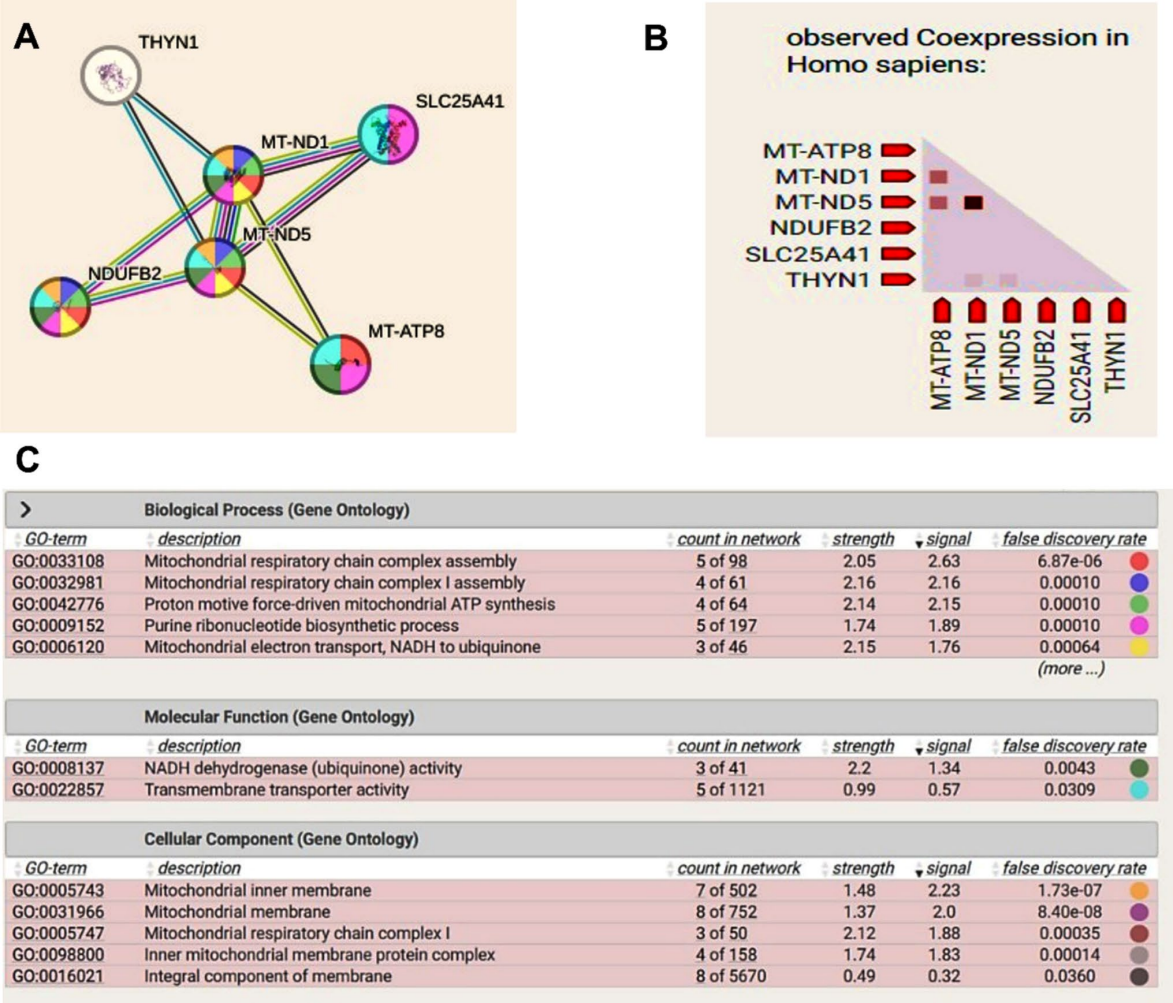
STRING database interaction for proteins related to mitochondrial function. **A** Protein–protein interaction analysis highlighting connections between mitochondrial proteins. **B** Coexpression analysis demonstrating the relationship among these proteins. **C** Gene ontology enrichment analysis, illustrating associated biological processes. In panel **A**, the colored lines represent different types of evidence for protein interactions: “red indicates fusion evidence, light blue represents database evidence, green denotes neighborhood interactions, blue indicates co-occurrence, purple represents experimental evidence, black represents coexpression, and yellow signifies text mining evidence”

The gene network analysis conducted using the “STRING database” highlights mitochondrial function, focusing on genes involved in the electron transport chain (ETC), ATP synthesis, and mitochondrial membrane integrity. Key genes such as *MT-ATP8*, *ND1*, *ND5*, *NDUFB2*, *SLC25A41*, and *THYN1* play crucial roles in these processes, which are essential for energy production and cellular metabolism. These genes exhibit strong interactions and coexpression, as demonstrated in Fig. 7A (Protein–Protein Interactions) and Fig. 7B (Coexpression Analysis).

Mitophagy is initiated when mitochondrial processes are compromised, such as through loss of membrane potential, oxidative stress, or impaired ATP production. The genes highlighted in the network are directly involved in maintaining mitochondrial integrity, as shown in Fig. 7C (Gene Ontology Analysis). Disruption of their function can lead to the accumulation of damaged mitochondria, signaling a need for mitophagic clearance to preserve cellular health.

## Discussion

HYP is a flavonoid with widely proven therapeutic and preventive uses (Xu et al. 2022). This work investigated the protective effect of HYP against MAL-induced neurotoxicity using differentiated human neuroblastoma cells as an in vitro model to evaluate the hypothesis. This neuronal secondary cell line was selected due to its human origin, avoidance of interspecies variation of data, and reasonable homogeneity of the cells’ population for reproducible data. MAL cytotoxic effect uses a wide range of concentrations from 0.01 to 100 mM to check for the EC50s by nonlinear curve fitting statistics. The reported human brain concentrations of MAL were reported to range from 0.25 to 1.2 mM (Jadhav et al. 1992). EC50 was estimated to be 2 mM, and the subsequent study assays were conducted using the estimated EC50 and one-tenth of EC50 (0.2 mM). Both concentrations cover the reported levels and have higher tested concentrations to get the toxic effect within the limited timeframe of in vitro assays. HYP protective effect was tested within the range of the previously published studies (Zhang and Zhang 2019; Qi et al. 2020).

The current data showed that MAL was cytotoxic to the tested neuronal cell line. HYP was shown to counteract MAL-induced neurotoxicity. Cotreatment of the cells with antioxidant glutathione or mitochondrial protective agent Co-Q10 showed that mitochondria are primary targets for MAL toxicity. MAL was shown to decrease ATP and MMP with decreased OCRs and increased lactate levels, and inhibition of mitophagy with reduced levels of PARKIN and PINK1 proteins. Krebs cycle assays showed that MAL decreased levels of PDH activities and α-KG. MCI and MCIII activities and kinetic assays showed that MAL significantly inhibited the MCI and MCIII activities. Q-PCR showed that MAL significantly decreased the expression of mitochondrial genes and mitophagy protein gene expression. In addition, MAL was shown to increase the MMF with increased unsaturated/saturated fatty acids ratios in the mitochondrial membranes. In addition, MAL was shown to increase the swelling of the mitochondrial membrane and increase membrane permeation of H^+^ and K^+^ ions. HYP was found to significantly alleviate the MAL effect on the neuronal cells’ mitochondria, with improved cell viability.

Our results demonstrate that hyperoside effectively mitigates malathion-induced mitochondrial dysfunction by restoring ATP production, mitochondrial membrane potential, and oxygen consumption rates while reducing oxidative stress and apoptosis. These findings align with prior studies that demonstrate malathion’s impact on mitochondrial function through oxidative phosphorylation disruption and ROS generation (Narasimhamurthy et al. 2024). Importantly, our bioinformatics analyses revealed that malathion exposure significantly affects pathways related to immune responses, apoptosis regulation, and mitochondrial function—key mechanisms implicated in neurodegenerative diseases such as Parkinson’s disease (Srivastava and Singh 2020; Narasimhamurthy et al. 2024). While these disruptions have been previously reported, our study adds value by integrating these findings with experimental evidence showing how hyperoside counteracts these effects at both cellular and molecular levels. The bioinformatics approach provides a broader context for interpreting our experimental results by identifying molecular pathways affected by malathion exposure. This system-level analysis highlights potential targets for therapeutic intervention beyond those identified through traditional biochemical assays alone. For example, the observed impact on immune response pathways suggests that malathion may exacerbate neuroinflammation, a hallmark of neurodegenerative diseases (Elmorsy et al. 2022). That hyperoside’s anti-inflammatory properties could play a critical role in its protective effects (Chen et al. 2022). Future studies should build upon these findings by employing advanced bioinformatics tools to elucidate further gene regulatory networks disrupted by malathion exposure. Additionally, integrating transcriptomic or proteomic data could provide deeper insights into how hyperoside modulates these pathways.

The effect of MAL on mitochondrial bioenergetics was previously reported in previous studies. Decreased ATP in MAL-treated cells was shown in previous investigations (Chan et al. 2006; Mostafalou et al. 2012; Shieh et al. 2019; Thosapornvichai et al. 2022). Karami-Mohajeri et al. (2014) reported that MAL (400 mg/kg) in rats induced a slight impairment in the function of complexes I, IV, and V, with no change in complex II. Interestingly, such a degree of insufficiency in respiratory chain enzyme activity can decrease the intracellular ATP. Also, MAL was shown to inhibit both MCI and MCIII activities in the rats’ brain regions. The disruption of cellular bioenergetics was highest within the hippocampus and corpus striatum brain regions by comparison to the cerebellum and cerebral cortex (Elmorsy et al. 2022).

Mitophagy is an evolutionarily conserved pathway by which cells get rid of their damaged, non-energizing mitochondria via lysosomal breakdown. Parkin and PINK1 proteins have been shown to play a major role in the mitophagy pathways (Rodriguez-Enriquez et al. 2006). Mitophagy eliminates damaged mitochondria, which can lead to increased release of ROS, activation of the NLRP3 inflammasome, as well as triggering apoptotic pathways (Murphy 2009; Heid et al. 2013; Palikaras et al. 2018; Youle 2019). The current data revealed that MAL significantly decreased both protein levels and the encoded gene expression. HYP significantly counteracted this action. Malathion was shown to inhibit mitophagy in the treated rats’ testicular tissue, which was shown by electron microscopy examination and lowered levels of PINK1, which is essential to direct the graded mitochondria to the lysosome (Omar et al. 2022).

The current data revealed that MAL significantly inhibited mitochondrial bioenergetics, while HYP was shown to counteract this MAL toxic effect significantly. This agrees with the previously published data (Li et al. 2023). In HT22 cells, HYP was demonstrated to lessen Aβ-induced mitochondrial dysfunction. Additionally, Li et al. showed that in 661 W photoreceptor cells, HYP attenuates oxidative stress-induced mitochondrial dysfunction and cell death. Combining these results, it is plausible that HYP protects photoreceptors in part by reducing mitochondrial damage brought on by oxidative stress. Additionally, it was demonstrated that HYP inhibits ischemia/reperfusion-induced oxidative stress and cell apoptosis by controlling the OMA1-OPA1 signaling pathway in an experimental model for ischemic acute kidney injury using both in vitro HK-2 cell line systems and in vivo Male C57BL/6 mice (Wu et al. 2019). In addition, HYP was reported to inhibit Amyloid β-protein (Aβ) 25–35-induced cytotoxicity and apoptosis in the primary cortical neurons by reversing Aβ-induced mitochondrial dysfunction, counteracting the effect on MMP decrease, ROS production, and mitochondrial release of cytochrome c (Zeng et al. 2011).

Mitochondrial impairment is widely accepted as the cornerstone of the different cytotoxic underlying mechanisms. Mitochondrial affection with altered electron transport chain disruption are the main sites for leakage of electrons, especially with inhibited MCI and MCIII, with the formation of ROS as superoxide anions, which then react with nitric oxide to produce the highly reactive peroxynitrites which were shown to play a major role in oxidative damage and genotoxicity (Liu et al. 2005). In addition, mitochondrial disruption causes leakage of cytochrome c to the cytoplasm and activation of the apoptotic pathways with induction of apoptosis (Kalpage et al. 2020). In addition, oxidative stress was proven to activate the apoptosis pathways, which was applied as an underlying mechanism in cancer oxidation therapy (Hanikoglu et al. 2020). As the pathways are complex and overlapping, we have used cotreatment with antiapoptotic (caspase-3 inhibitor z-VAD-fmk), antioxidant (reduced glutathione), and mitochondrial protective Co-Q10. MTT showed that mitochondrial protection showed the highest protective effect against MAL toxicity, which highlights mitochondria as a primary target for MAL toxicity in the differentiated neuroblastoma cell line.

## Study limitations

The current study evaluated the protective effect of HYP-induced toxicity via bioenergetics disruption in the differentiated SH-SY5Y neuronal cell line via different assays, which support the conclusion that HYP is cytoprotective for the neuronal cell line. However, the study has its limitations as being an in vitro study, which may not resemble the normal in vivo micro-environment interactions among the pathways. To enhance the translational value of this study, future research should consider an in vivo model to validate the observed protective effects of hyperoside in a more physiologically relevant setting. Such studies could involve animal models of Parkinson’s disease or malathion toxicity, assessing behavioral outcomes, dopamine levels, and mitochondrial function in brain tissue. This would provide valuable insights into the potential neuroprotective effects of hyperoside in a complex biological system. Secondly, not all pathways were investigated, which can be further assessed in subsequent studies covering mitophagy and autophagy pathways key proteins, such as LC3-II, Beclin-1, P62, BNIP3, and FUNDC1, as well as the other interconnecting cytotoxicity mechanisms related to the mitochondrial inhibition, such as apoptosis, oxidative stress and inflammation. The study’s immortalized cell line may go through multiple in vitro cycles of dedifferentiation, which could affect the original tissues’ requirements and how they react to toxins. However, the study was based on passage five of the cell line, and the secondary cell line was used to get a sufficient number of cells for the different assays with reproducible data. In addition, the human cells were preferred over the animal’s isolated primary cell line to overcome the interspecies difference effect on the assay outcomes. While our in vitro model provides valuable mechanistic insights, it is important to acknowledge that it may not fully capture the complexity of the brain environment, including factors such as blood–brain barrier penetration, glial cell interactions, and systemic metabolic influences. These aspects could be addressed in future in vivo studies. Finally, further studies are recommended using in vivo models and checking for further subcellular pathways to support the study findings. These could include investigating the effects of hyperoside on neuroinflammation, synaptic plasticity, and long-term neuroprotection in animal models of neurodegenerative diseases.

## Conclusions

The current study evaluated the protective effect of HYP against MAL-induced bioenergetic disruption in differentiated human neuroblastoma cells. Our findings demonstrated that HYP provided significant cryoprotection against MAL-induced bioenergetic impairments across various conditions. These results support the potential therapeutic role of HYP in mitigating neurotoxicity associated with MAL and other related pesticides, which are known to cause neurodegenerative disorders through mitochondrial toxicity in neuronal tissues.

## Supplementary Information

Below is the link to the electronic supplementary material.

Supplementary file1 (PDF 682 KB)

## Data Availability

The data supporting the results of this study can be obtained from the corresponding authors upon reasonable request.
