# Supplementary material for: Hyperoside flavonoids protect against malathion-induced mitochondrial toxicity in the differentiated SH-SY5Y cells
Source: Naunyn Schmiedebergs Arch Pharmacol. 2025 Jun 25;398(12):17803–21. doi: 10.1007/s00210-025-04379-2 (PMC12678533; doi:10.1007/s00210-025-04379-2)
Supplement: Supplementary file 1 — Supplementary file1 (PDF 682 KB) [file 210_2025_4379_MOESM1_ESM.pdf]

Table S1: Components of different solutions used in this work

Solution	Contents
Modified Hank's solution	5.6 mM KCl, 138 mM NaCl, 4.2 mM NaHCO3, 1.2 mM NaH2PO4, 2.6 mM CaCl2, 1.2 mM MgCl2, 10 mM HEPES (pH 7.4 with NaOH), and 0.1% (w/v) bovine serum albumin (BSA).
MCI assay buffers	For 10 mL of complex I assay buffer, the following chemicals were used: 5 mL of potassium phosphate buffer (50 mM pH 7.6), 2 mL of dichloroindophenol sodium salt hydrate (DCIP) stock (0.6 mM), 140 µL of decylubiquinone (DUB) (5 mM), and 10 µL of antimycin A (1 mM). The volume was adjusted to 10 mL with distilled water, and then fatty acid-free bovine serum albumin (BSA) (35 mg) was added.
MCIII assay buffer	For each 10 mL of mitochondrial complex III buffer, the following chemicals were used: 500 µL of potassium phosphate buffer (0.5 M, pH 7.5), 750 µL of oxidized cytochrome c, 400 µL of sodium azide (10 mM), 20 µL of ethylenediaminetetraacetic acid (EDTA) (5 mM, pH 7.5), and 10 µL of Tween-20 (2.5%) (*v*/*v*). The final volume was adjusted to 10 mL with distilled water.
Solution A	0.2 M sucrose, 1 mM Tris, 1 mM Mops, 10 µM EGTA, 1 mM Na_3PO_4, 3 $\mu g.ml^{-1}$ oligomycin, 5 mM succinate, and 2 µM rotenone
Solution B	0.135 M CH_3COOK, 5 mM HEPES, 0.1 mM EGTA, 2 µM rotenone, 1 $\mu g.ml^{-1}$ valinomycin, and 0.2 mM EDTA
Solution C	0.135 M KNO_3, 5 mM HEPES, 0.1 mM EGTA, and 0.2 mM EDTA

Table S2: Two-way ANOVA analysis regarding the effect of malathion (MAL) and hyperoside (HYP) on mitochondrial swelling and membrane permeation to hydrogen and potassium ions

Assays	0.2 mM			2 mM		
	Source of Variation	% of total variation	*p*-value	Source of Variation	% of total variation	*p*-value
Swelling	Interaction	14.61	< 0.0001	Interaction	14.88	< 0.0001
	Treatment	46.31	< 0.0001	Treatment	30.08	< 0.0001
	Time	35.27	< 0.0001	Time	49.53	< 0.0001
K^+ permeation	Interaction	11.31	< 0.0001	Interaction	11.33	< 0.0001
	Treatment	33.12	< 0.0001	Treatment	30.20	< 0.0001
	Time	47.87	< 0.0001	Time	46.93	< 0.0001
H^+ permeation	Interaction	11.58	< 0.0001	Interaction	12.47	< 0.0001
	Treatment	24.23	< 0.0001	Treatment	21.72	< 0.0001
	Time	59.96	< 0.0001	Time	61.74	< 0.0001

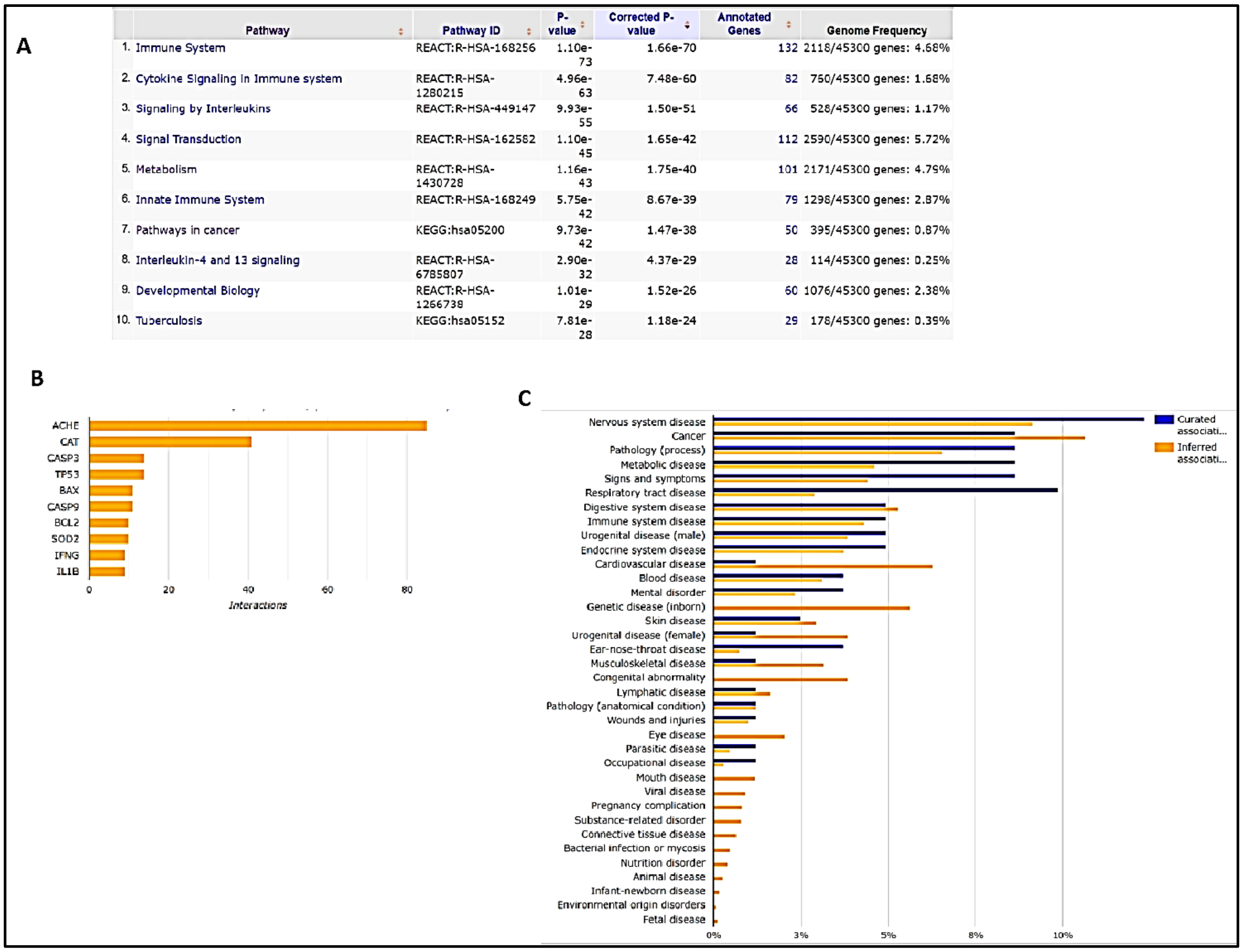

A

	Pathway	Pathway ID	P-value	Corrected P-value	Annotated Genes	Genome Frequency
1.	Immune System	REACT:R-HSA-168256	1.10e-73	1.66e-70	132	2118/45300 genes: 4.68%
2.	Cytokine Signaling in Immune system	REACT:R-HSA-1280215	4.96e-63	7.48e-60	82	760/45300 genes: 1.68%
3.	Signaling by Interleukins	REACT:R-HSA-449147	9.93e-55	1.50e-51	66	528/45300 genes: 1.17%
4.	Signal Transduction	REACT:R-HSA-162582	1.10e-45	1.65e-42	112	2590/45300 genes: 5.72%
5.	Metabolism	REACT:R-HSA-1430728	1.16e-43	1.75e-40	101	2171/45300 genes: 4.79%
6.	Innate Immune System	REACT:R-HSA-168249	5.75e-42	8.67e-39	79	1298/45300 genes: 2.87%
7.	Pathways in cancer	KEGG:hsa05200	9.73e-42	1.47e-38	50	395/45300 genes: 0.87%
8.	Interleukin-4 and 13 signaling	REACT:R-HSA-6785807	2.90e-32	4.37e-29	28	114/45300 genes: 0.25%
9.	Developmental Biology	REACT:R-HSA-1266738	1.01e-29	1.52e-26	60	1076/45300 genes: 2.38%
10.	Tuberculosis	KEGG:hsa05152	7.81e-28	1.18e-24	29	178/45300 genes: 0.39%

Figure S1. Malathion impact from comparative toxicogenomics databases. A) Top pathway that malathion has been implicated in its dysregulation. **B)** Top genes that have shown to be affected by malathion. **C)**Top diseases related to malathion toxicity.

A

	Disease	Direct Evidence	Enrichment Analysis	Inference Network	Inference Score
1.	Brain Ischemia			*10 genes* CASP3 | CAT | CYCS | MAPK1 | MAPK3 | NFKBIA | PARP1 | RELA | TNF | TNFRSF1A	39.56
2.	Alzheimer Disease			*6 genes* ACHE | BAX | BCL2 | CASP3 | ESR1 | TNF	19.80
3.	Brain Injuries, Traumatic			*3 genes* PARP1 | RELA | TNF	15.71
4.	Trigeminal Neuralgia			*3 genes* MAPK1 | MAPK3 | TNF	14.49
5.	MIGRAINE WITH OR WITHOUT AURA, SUSCEPTIBILITY TO, 1			*2 genes* ESR1 | TNF	12.72
6.	Subarachnoid Hemorrhage			*3 genes* CASP3 | RELA | TNF	11.89
7.	Brain Neoplasms			*3 genes* RELA | SIRT1 | SMO	11.21
8.	Muscular Dystrophies			*2 genes* ACHE | PRIMA1	10.73
9.	Ceroid lipofuscinosis, neuronal 1, infantile			*2 genes* PARP1 | SIRT1	10.46
10.	Ceroid Lipofuscinosis, Neuronal, 6			*2 genes* MAPK1 | MAPK3	9.91

B

	Pathway	Pathway ID	P-value	Corrected P-value	Annotated Genes	Genome Frequency
1.	Apoptosis	KEGG:hsa04210	3.08e-22	1.60e-19	12	138/45300 genes: 0.30%
2.	Hepatitis B	KEGG:hsa05161	1.31e-17	6.85e-15	10	144/45300 genes: 0.32%
3.	Pathways in cancer	KEGG:hsa05200	1.14e-16	5.93e-14	12	395/45300 genes: 0.87%
4.	Toxoplasmosis	KEGG:hsa05145	1.81e-16	9.41e-14	9	113/45300 genes: 0.25%
5.	AGE-RAGE signaling pathway in diabetic complications	KEGG:hsa04933	8.74e-15	4.55e-12	8	99/45300 genes: 0.22%
6.	Amyotrophic lateral sclerosis (ALS)	KEGG:hsa05014	9.79e-15	5.10e-12	7	51/45300 genes: 0.11%
7.	Tuberculosis	KEGG:hsa05152	1.18e-14	6.13e-12	9	178/45300 genes: 0.39%
8.	TNF signaling pathway	KEGG:hsa04668	1.79e-14	9.31e-12	8	108/45300 genes: 0.24%
9.	Colorectal cancer	KEGG:hsa05210	3.25e-14	1.69e-11	7	60/45300 genes: 0.13%
10.	Proteoglycans in cancer	KEGG:hsa05205	3.89e-14	2.03e-11	9	203/45300 genes: 0.45%

Figure S2. HYP impact based on comparative toxicogenomics databases: A) Top diseases that can be prevented or treated with HYP. **B)** Top pathways that have been shown to be affected by the protective role of HYP.
